# Combinational Pretreatment of Colony-Stimulating Factor 1 Receptor Inhibitor and Triptolide Upregulates BDNF-Akt and Autophagic Pathways to Improve Cerebral Ischemia

**DOI:** 10.1155/2020/8796103

**Published:** 2020-10-31

**Authors:** Xiaoxue Du, Feng Gao, Shijia Chen, Benson O. A. Botchway, Nashwa Amin, Zhiying Hu, Marong Fang

**Affiliations:** ^1^Institute of Neuroscience, School of Medicine, Zhejiang University, Hangzhou, China; ^2^Translation Medicine Center, Affiliated Hangzhou First People's Hospital, School of Medicine, Zhejiang University, Hangzhou, China; ^3^Department of Neurology, The Children's Hospital, Zhejiang University School of Medicine, National Clinical Research Center for Child Health, Hangzhou, China; ^4^Department of Zoology, Faculty of Science, Aswan University, Egypt; ^5^Department of Obstetrics and Gynecology, Hangzhou Red Cross Hospital, Zhejiang Integrated Traditional and Western Medicine Hospital, Hangzhou, China

## Abstract

Ki20227, a selective inhibitor of colony-stimulating factor 1 receptor (CSF1R), has been suggested to regulate microglia inflammatory function and neuronal synaptic plasticity. Triptolide (TP) pretreatment has neuroprotective effects through its anti-inflammatory and antiapoptotic features in ischemic stroke mice. However, the underlying mechanism and pathway are presently unclear. We thus investigated the association between neuroprotective effects of combined TP and Ki20227 and BDNF-Akt and autophagy pathways. Ki20227 was administrated for 7 days, and TP was administered once 24 hours prior to building the ischemic stroke model in C57BL/6 mice. Behavioral tests, Golgi staining, immunofluorescence, and western blot analyses were employed to examine neuroprotective effects of TP and Ki20227. TP and Ki20227 pretreatments improved the neurobehavioral function in stroke mice. Synaptic protein expressions and density of dendritic spine density were upregulated in Ki20227 and TP pretreated stroke mice. Further, optimized integration of TP and Ki20227 pretreatments upregulated the NeuN expression and downregulated Iba1 expression after stroke. In addition, both TP and Ki20227 pretreatments significantly upregulated BDNF, p-Akt/Akt, and Erk1/2 protein expressions and autophagy related proteins (LC3II/I, Atg5, and p62), indicating the activation of BDNF and autophagic pathways. Optimized integration of TP and Ki20227 can improve cerebral ischemia by inhibiting CSF1R signal and trigger autophagy and BDNF-Akt signaling pathways to increase dendritic spine density and synaptic protein expressions, which in turn enhances neurobehavioral function.

## 1. Introduction

Ischemic stroke is a serious neurological disease that has high incidence, mortality, and disability rates and endangers both the physical and mental health of patients without effective interventions [1, 2]. Several investigative studies are targeting inflammatory-related factors to alleviate cerebral ischemic injury by reducing the area of cerebral infarction and improve neurological deficits [3].

Colony-stimulating factor 1 receptor (CSF1R), a transmembrane tyrosine kinase, is involved in the proliferation and differentiation of macrophages/microglia in the brain [4]. The inhibition of CSF1R signaling has a neuroprotective role in several diseases, including ischemic stroke [5, 6]. Ki20227, as a selective inhibitor of CSF1R, inhibits the phosphorylation of CSF1R [7]. Notably, an *in vitro* study showed Ki20227 to inhibit the normal growth of m-nfs-60 cells [8]. Similarly, animal studies evidenced that microglia density was reduced after continuous administration of Ki20227, with both neurobehavioral function and neuron synaptic plasticity also affected [9]. Nonetheless, the underlying mechanism pertinent to Ki20227 on neuronal recovery after cerebral ischemia is unclear.

Triptolide (TP) is a major active component of the traditional Chinese herb *Tripterygium wilfordii* Hook.f, possesses anti-inflammatory and immunosuppressive effects, and was reported to improve the survival rate of allograft [10]. In an animal model of middle cerebral artery occlusion (MCAO), TP pretreatment reduced the infarct area and suppressed iNOS and NF-*κ*B expressions to alleviate microglia-mediated neuroinflammation [11]. Our previous studies showed TP pretreatment to regulate autophagy, oxidative stress, and PI3K/Akt/mTOR pathways in an MCAO rat model [12].

The brain-derived neurotrophic factor (BDNF) has been implicated in ischemic stroke pathogenesis. Particularly, BDNF is involved in both MAPK/ERK1/2 and PI3K complex pathways and is central in regulating cell growth, proliferation, and survival and axonal sprouting, which are important for poststroke functional recovery [13]. In an animal mode of ischemia, the upregulation of the BDNF/TrkB expression altered that microglia state to improve injury [14].

Autophagy deficiency is involved in the pathogeneses of neurodegenerative diseases [15]. Autophagy is not only impaired in neurons, glia cell, and brain microvascular cells by focal cerebral ischemia but also exert “good cop/bad cop” functions at different times in the wake of ischemic damage [16]. The potential therapeutic applications of pharmacological modulators of autophagy include some naturally occurring polyphenolic compounds, such as TP. TP can enhance mitochondrial function and attenuate autophagic processes in stroke [17].

The underlying mechanism pertinent to Ki20227 on neuronal recovery after cerebral ischemia is unclear. Similarly, neither the combined treatment of TP and CSF1R inhibitor in cerebral ischemia nor its relationship to BDNF/autophagic pathways in the stroke model has been explored. However, with TP and Ki20227 individually possessing neuroprotective features, we aimed to investigate the therapeutic effect following the combined administration of these two medicinal agents in cerebral ischemic stroke. Our results showed that Ki20227 and TP cotreatment could alleviate ischemic injury by upregulating the BDNF/Akt/Erk1/2 and autophagy pathways.

## 2. Material and Methods

### 2.1. Animal Preparation

We performed this study following the Guide for Care and Use of Laboratory Animal Center of Zhejiang University, with animal treatments approved by the Ethics Committee for Use of Experimental Animals in Zhejiang University. The ethics protocol number (ZJU20160267) of this study was provided by the Animal Center of Zhejiang University. Male C57BL/6 mice (weighing 20–30 g) were purchased from the Zhejiang Medical Academy Laboratory. Thy-YFP mice purchased from Jackson Animal Centre (no.: 003782) were used for confirming the location of the ischemic penumbra. All animals were kept in pathogen-free separated clean cages, with a constant temperature of 24°C under a 12 h light/dark cycle. Animals had free access to food and water.

### 2.2. Experimental Groups and Drug Administration

Ninety male mice were randomly divided into six experimental groups: C57BL/6 mice (WT group), C57BL/6 cerebral ischemic mice (WT+stroke group), C57BL/6 cerebral ischemic mice with TP pretreatment (WT+stroke+TP), C57BL/6 mice with Ki20227 pretreatment (WT+K), C57BL/6 cerebral ischemic mice with Ki20227 pre-treatment (WT+stroke+K), and C57BL/6 cerebral ischemic mice with Ki20227 and TP pretreatments (WT+stroke+K+TP). Ki20227 was administered by oral gavage at a dose of 0.002 mg/kg/day for 7 or 14 days [6]. After 7 days of Ki20227 administration, a cerebral ischemia mouse model was established in stroke groups, and Ki20227 administration was continued for the next 24 hours. TP was dissolved in 0.1% DMSO salt solution and stored at 4°C in the dark. TP was intraperitoneally injected once at a dose of 0.2 mg/kg 24 hours before establishing the cerebral ischemic model [12]. The dead or incapacitated mice were excluded in accordance with animal ethical requirements.

### 2.3. Establishment of Cerebral Ischemic Mouse Model

The cerebral ischemic mouse model was established as previously described [18]. Rose Bengal dye (Sigma-Aldrich, # 330000) was dissolved in 0.1 M PBS solution with 10 mg/mL final concentration after filtering through 0.45 m filters and placed in the dark until use. In a building stroke model, mice were intraperitoneally injected with Rose Bengal dye (100 mg/kg) before anaesthetizing with inhaled isoflurane (RWD, China). After 5 minutes, a flexible cool light guide attached to a light-emitting diode cold light source (OPLENIC, M-IL-HAL 3001) was positioned above the target area that controlled the right forelimb motor cortex (1.75 mm lateral to Bregma, +0.5 mm, left hemisphere). To induce focal cerebral ischemia, the target area was illuminated for 15 minutes before suturing the wound. Mice were placed on a warm blanket until they woke up.

### 2.4. Behavioral Tests

#### 2.4.1. Rotarod Test

The rotarod test was used to measure motor coordination and antifatigue ability of mice before and after cerebral ischemia. It (i.e., rotarod test) consisted of a roller with a diameter of 3 cm and length of 6 cm, 4 tracks with a width of 6 cm for each track, infrared detector, and a computer. The infrared sensor measured the duration mice stayed on the stick and the movement speed of mice when they fell. The total program time was set to 300 seconds, and the speed was accelerated from 8 to 40 rpm (revolutions per minute). Each group of mice was trained thrice a day, with a resting period of 10 minutes. Mice that stayed on the rotating rod instrument for more than 200 seconds were used to establish the stroke model. The duration of each group of mice on the rotating rod was recorded in two days before and one day after establishing the stroke model.

#### 2.4.2. Neurobehavioral Score

The neurological scores were evaluated as previously described [19]. Neurological function assessments were scored on five levels, with 0 implying no observable deficits, 1 representing forelimb flexion, 2 indicating forelimb flexion plus decreased resistance to lateral push, 3 corresponding to unidirectional circling, and 4 denoting unidirectional circling plus decreased level of consciousness. The above behavioral observations were carried out in a blinded process.

### 2.5. TTC Staining

TTC staining was employed to ascertain successfulness of the ischemia mouse model. Each fresh and frozen brain was cut at 2 mm intervals from the frontal pole using mouse brain matrix. Sections were incubated with 2% 2,3,5-triphenyltetrazolium chloride (TTC, Sigma-Aldrich Co., USA) at 37°C for 10 minutes in the dark, and then fixed in 4% paraformaldehyde overnight, and photographed. The unstained areas were considered to be the infract areas.

### 2.6. Golgi Staining

Golgi staining followed a previously described method [20]. Brains from each group of mice were perfused with 0.9% NaCl solution, fixed in 0.5% paraformaldehyde PBS solution, transferred into Golgi-Cox solution afterwards, and incubated below 37°C in darkness for 6 days. After dehydrating in 30% sucrose in PBS before sinking, brains were sectioned at 200 *μ*m by vibratome (Leica, VT1200, Germany). After rinsing twice for 5 minutes with distilled water, brain slices were dehydrated in 50% ethanol for 5 minutes before transferring to 75% ammonia solution for about 10 minutes. They were then kept in 5% sodium thiosulfate for 10 minutes in the dark and dehydrated through 50%, 50%, 70%, 80%, 95%, 100%, and 100% ethanol afterwards. Following dimethylbenzene transparency, brain slices were covered with a neutral resin. Spine density measurement was done by selecting a minimum of five pyramidal neurons from each mouse. Matching sections of distal branch dendrites were photographed using X100 oil objective lens for counting of spine numbers in 50 *μ*m segments, with results expressed as number of spines per 10 *μ*m. Two experimental blinds to the experimental groups were performed to determine spine quantification. Counting of spines was manually done on randomly selected second-order segments from the apical dendrites. Approximately, 2–3 segments were counted per slide with no less than 6 slides being used for each mouse.

### 2.7. Immunofluorescence Staining

Mouse brains were perfused with 0.9% NaCl solution, fixed in 4% paraformaldehyde PBS solution, and dehydrated in 30% sucrose solution. After embedding, brain sectioning was done coronally. Primary antibodies mouse anti-Iba1 (1 : 500; Abcam Cat# ab15690, RRID:AB_2224403), and mouse anti-NeuN (1 : 100; Abcam Cat# ab104224, RRID:AB_10711040) were applied. Secondary antibodies, goat anti mouse Alexa Fluor 488 or 594 (1 : 500, EARTHOX, San Francisco, # E031220-01), were employed. Antiquenching mounting medium containing DAPI (VECTASHIELD, USA) was added to slides prior to being covered with coverslips for observation. Positive cells were detected by a fluorescence microscope (Olympus BX51, NIKON, Japan) at excitation/emission wavelengths of 547/570 nm (Cy3, red), 488/520 nm (FITC, green), and 360/460 nm (FITC, blue). Images were taken under 400x magnifications in 5-vision fields/section randomly, and the immunoreactive cells were counted.

### 2.8. Western Blot

Total proteins of brain tissues were extracted by using RAPI buffer with protease inhibitor cocktail EDTA-free (Rocher, Switzerland) and phosphate inhibitors (Rocher, Switzerland). Protein concentrations were measured with BCA kits. Protein (30 *μ*g) sample from each group was subjected to electrophoresis on 12% SDS-PAGE gel. Running process involved 70 V for 30 minutes and 100 V for 100 minutes. Proteins were transferred to the PVDF membrane with Bio-Rad TransBlot apparatus for 120 minutes. The PVDF membranes were blocked in 5% BSA solution for 2 hours at room temperature. Primary antibodies, rabbit anti PSD95 (1 : 1000, Abcam Cat# ab18258, RRID:AB_444362), SYN (1 : 1000, Abcam Cat# ab32127, RRID:AB_2286949), MAP-2 (1 : 1000, Abcam Cat# ab5392, RRID:AB_2138153), GADPH (1 : 1000, Abcam Cat# ab181602, RRID:AB_2630358), LC3B (1 : 1000, Abcam Cat# ab48394, RRID:AB_881433), Atg5 (1 : 1000, Abcam Cat# ab108327, RRID:AB_2650499), P62 (1 : 1000, Abcam Cat# ab109012, RRID:AB_2810880), Beclin1 (1 : 1000, Abcam Cat# ab210498, RRID:AB_2810879), BDNF (1 : 1000, Abcam Cat# ab108319, RRID:AB_10862052), Akt (1 : 1000, Abcam Cat# ab8805, RRID:AB_306791), p-Akt (1 : 1000, Abcam Cat# ab38449, RRID:AB_722678), Erk1/2 (1 : 1000, Abcam Cat# ab17942, RRID:AB_2297336), and CSF1R (1 : 1000, Abcam Cat# ab254357), were incubated overnight at 4°C. The secondary antibody HRP-conjugated goat anti-rabbit (1 : 5000; EarthOx, USA) was incubated for 2 hours after the primary antibody had been washed thrice with TBST solution for 15 minutes (5 minutes each time). The enhanced chemiluminescence (ECL) (Thermo Fisher Scientific, USA) was used for analyzing protein detection. Imaging and quantification of protein bands were proceeded with ImageJ software. Each experiment was triplicated.

### 2.9. Statistical Analysis

Band intensity of western blot results were calculated using ImageJ. Glia staining and immunofluorescence were analyzed by Image-Pro Plus software. Statistical analysis was performed using SPSS 20.0 software. The difference was evaluated by one-way ANOVA with Tukey's tests between drug treatment and control group in stroke models. Regarding the data of multiple groups in the rotarod test, the difference was analyzed using the two-way ANOVA with Bonferroni's posttest. Values are presented as means ± SEM. For all tests, three levels of significance were determined: ^∗^*P* < 0.05, ^∗∗^*P* < 0.01, and ^∗∗∗^*P* < 0.001, where “^∗^” was the difference between the WT and WT+stroke groups, “^#^” was the difference between the WT+stroke and WT+stroke+K or WT+stroke+TP groups, “^&^” was the difference between the WT+stroke+K and WT+stroke+TP groups, and “^^^” was the difference between the WT+stroke+K+TP and WT+stroke+K or WT+stroke+TP groups.

## 3. Results

### 3.1. Ki20227 Administration Reduces Microglia Number

**Figure 1:**
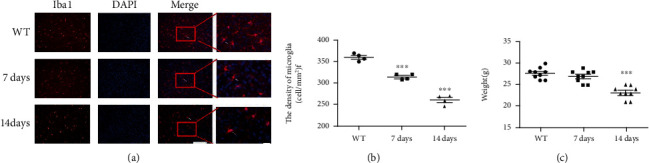
Ki20227 administration reduces microglia number. (a) Immunofluorescence staining images of Iba1 in the cortex of WT group and Ki20227-treated mice on days 7 and 14. Scale bar = 100 *μ*m and 50 *μ*m. Merged figure shows positive cells (white arrow). (b) Quantitative analyses of microglia density. WT group vs. Ki20227 administration on day 7, ^∗∗∗^*P* < 0.001; WT vs. Ki20227 administration on day 14, ^∗∗∗^*P* < 0.001; two-way ANOVA with Bonferroni's posttest, *n* = 4. Data were presented as mean ± SEM. (c) Quantitative analyses of weight changes after Ki20227 administration. Data were presented as mean ± SEM. One-way ANOVA with Tukey's tests, *n* = 7. No significant difference between WT and Ki20227 on day 7. WT vs. Ki20227 on day 14, ^∗∗∗^*P* < 0.001.

In order to study the time-dependent effect of Ki20227 on the number of microglia, assessments were done 1 day prior to Ki20227 administration, as well as 7 and 14 days after Ki20227 administration. Iba1 immunofluorescence staining results showed decreased microglia number in a time-dependent manner after administration (Figure 1(a)). Microglia number was considerably reduced on days 7 and 14 in the cerebral cortex of WT mice after Ki20227 treatment when compared with the control group (Figure 1(b), ^∗∗∗^*P* < 0.001). Further, mouse weights were measured after Ki20227 administration. On day 14 following Ki20227 administration, lost weight in mice were pronounced (Figure 1(c), ^∗∗∗^*P* < 0.001). Owing to these drug effects, day 7 after Ki20227 administration was used as the key time point for subsequent experiments.

### 3.2. Ki20227 and TP Improve Behavioral Function in Cerebral Ischemia

**Figure 2:**
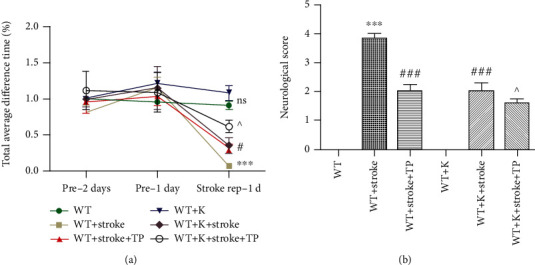
TP and Ki20227 enhance behavioral function. (a) Graph represents the total time difference of experimental groups at pre-1 day, pre-2 days, and stroke rep-1 day. *F* values were, respectively, pre-1 day, pre-2 days. and stroke-rep 1 day. Data were expressed as mean ± SEM. No significant difference between the WT and WT+K groups; the WT group vs. WT+stroke group, ^∗∗∗^*P* < 0.001; the WT+stroke group vs. WT+stroke+TP group and WT+stroke+K group, ^#^*P* < 0.05; the WT+stroke+K+TP group vs. WT+stroke+TP group and WT+stroke+K group, ^^^*P* < 0.05. Data were presented as mean ± SEM. Two-way ANOVA with Bonferroni's posttest, *n* = 11. (b) Quantitative analyses of neurological score of experimental groups at 1 day after stroke. Data were expressed as mean ± SEM. One-way ANOVA with Tukey's tests, *n* = 12. The WT vs. WT+stroke and WT+K vs. WT+stroke+K groups, ^∗∗∗^*P* < 0.0001; the WT+stroke vs. WT+stroke+TP and WT+stroke+K groups, ^###^*P* < 0.0001; and the WT+stroke+TP and WT+stroke+K vs. WT+stroke+K+TP, ^^^*P* < 0.05.

The total time that each mouse stayed on the rotating rod depicted the physical function. The average time difference that each group of mice stayed on the rotating rod in pre-2 day, pre-1 day, and stroke-rep 1 day is shown in Figure 2(a). No significant difference in the average time was discerned between the WT and WT+K groups (*P* > 0.05), indicating that Ki20227 administration had no effect on fatigue tolerance and balance ability of mice. After cerebral ischemia, the total time spent on the rotating rod in the WT+stroke group was significantly decreased (^∗∗∗^*P* < 0.01), while increase in the WT+stroke+TP and WT+stroke+K groups was pronounced (^#^*P* < 0.05). Similarly, time spent on the rotating rod by the WT+stroke+K+TP group was increased when compared with both the WT+stroke+TP and WT+stroke+K groups (^^^*P* < 0.05).

In the neurological score, each group of mice was subjected to neurobehavioral scoring in a double-blinded manner at 24 hours following cerebral ischemia (Figure 2(b)). Neurobehavioral scores of the WT+stroke group was considerably increased when compared with the WT group (^∗∗∗^*P* < 0.001). However, neurobehavioral scores of both the WT+stroke+K and WT+stroke+TP groups were decreased in comparison with the WT+stroke group (^###^*P* < 0.001). In the WT+stroke+K+TP group, decreased neurobehavioral scores was observed when compared with the WT+stroke+K and WT+stroke+TP groups (^^^*P* < 0.05).

### 3.3. Ki20227 and TP Pretreatments Upregulate NeuN Expression and Downregulate Iba1 Expression in the Cortical Penumbra in Cerebral Ischemia

**Figure 3:**
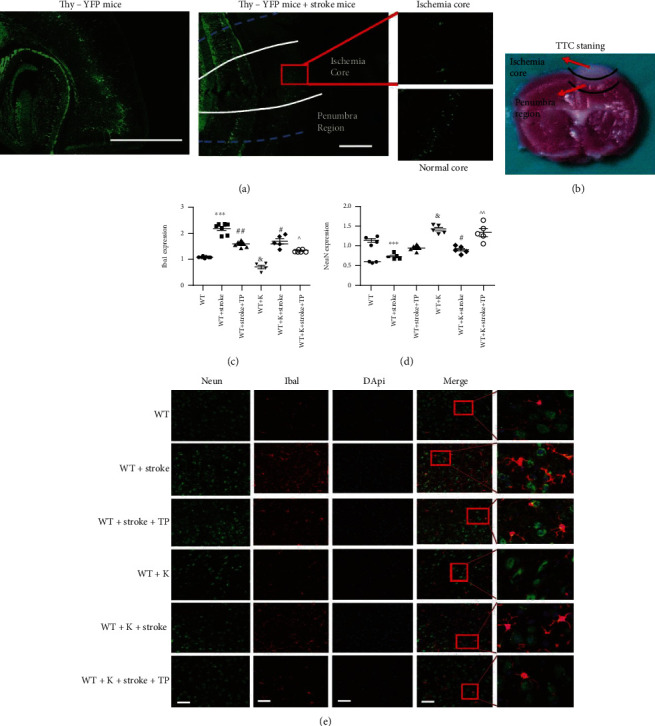
Optimized integration of TP and Ki20227 pretreatments correspondingly increases and decreases NeuN and Iba1 expressions in the cortical penumbra. (a) Thy-YFP mice were employed to show the ischemia core and penumbra region. We counted fifty YFP-positive cells and axons in the pyramidal neurons. After stroke, YFP-positive cell deaths with fluorescence were suppressed in similitude to the normal core region. Scale bar = 100 *μ*m and 50 *μ*m. (b) TTC staining 24 hours after ischemia showing the ischemic core and peri-infarct region. The pink area between the normal cortex and ischemic core was the penumbra region. (c) Quantitative analyses of Iba1 expression. (d) Quantitative analyses of NeuN expression. (e) Immunofluorescence staining images of experimental groups. Scale bar = 20 *μ*m. Data were expressed as mean ± SEM. One-way ANOVA with Tukey's tests, *n* = 5. The WT vs. WT+stroke groups, ^∗∗∗^*P* < 0.0001; the WT+K vs. WT+stroke+K groups, ^∗∗∗^*P* < 0.0001; the WT+stroke group vs. WT+stroke+TP and WT+stroke+K groups, ^#^*P* < 0.05, ^##^*P* < 0.01; the WT+K group vs. WT group, ^&^*P* < 0.05; and the WT+stroke+K+TP group vs. WT+stroke+TP and WT+stroke+K groups, ^^^*P* < 0.05, ^^^^*P* < 0.01.

Different cell types, such as neurons, microglia, and astrocytes, are connected to each other in the brain. The brain maintains functional working capacity through signal and synaptic transductions. Thy-YFP mice were employed to evidence the neuronal change in both ischemic and normal core sections (Figure 3(a)). TTC staining showed the ischemia core and penumbra regions (Figure 3(b)). NeuN and Iba1 expressions correspondingly represented neuronal and microglia changes after stroke (Figures 3(c) and 3(d)). NeuN expression in the ischemic penumbra of the cerebral cortex was significantly reduced, while Iba1 expression was augmented when the WT+stroke group was compared with the WT group (^∗∗∗^*P* < 0.001). Following treatments, Iba1 expression in both the WT+stroke+TP and WT+stroke+K groups were significantly decreased in comparison with the WT+stroke group (^#^*P* < 0.05, ^##^*P* < 0.01). There was no significant difference between the WT+K and WT groups concerning Iba1 expression.

In the NeuN expression, we observed increased intensity in the WT+K group when compared with the WT group (^&^*P* < 0.05). In comparison with the WT+stroke group, NeuN expression in the WT+stroke+K group was significantly increased (^#^*P* < 0.05). Further, NeuN and Iba1 expressions were increased and decreased, respectively, in the WT+stroke+K+TP group when compared with both the WT+stroke+TP and WT+stroke+K groups (^^^*P* < 0.05, ^^^^*P* < 0.01).

### 3.4. Ki20227 and TP Decrease CSF1R Protein Expression in Cerebral Ischemia

**Figure 4:**
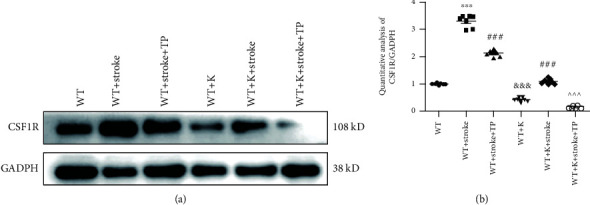
TP and Ki20227 downregulate CSF1R protein expression. (a) Western blot images of CSF1R and GADPH protein expressions of experimental groups. (b) Quantitative analyses of CSF1R protein expression. Data were expressed as mean ± SEM. One-way ANOVA with Tukey's tests, *n* = 7. The WT vs. WT+stroke groups, ^∗∗∗^*P* < 0.001; the WT+Stroke+TP and WT+K+stroke groups vs. WT+stroke group, ^###^*P* < 0.001; the WT+stroke+K+TP group vs. WT+stroke+TP and WT+stroke+K groups, ^^^^^*P* < 0.001.

CSF1R protein expression was used to ascertain the inhibitory effects of Ki20227 and TP (Figure 4). In the WT+K group, CSF1R expression was significantly reduced when compared with the WT group (^&&&^*P* < 0.001). The upregulation of CSF1R expression in the WT+stroke group in comparison with the WT group indicated CSF1R signal activation (^∗∗∗^*P* < 0.001). However, CSF1R protein expression in both the WT+stroke+K and WT+stroke+TP groups was lower than that of the WT+stroke group (^###^*P* < 0.001). Also, CSF1R expression was further downregulated in the WT+K+stroke+TP group when compared with both the WT+stroke+TP and WT+stroke+K groups (^^^^^*P* < 0.001).

### 3.5. Optimized Integration of Ki20227 and TP Pretreatments Improves Dendritic Spine Density in the Cortical Penumbra of Cerebral Ischemia

**Figure 5:**
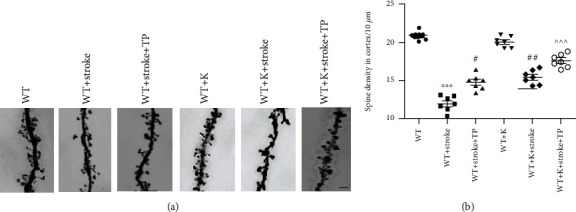
Optimized integration TP and Ki20227 pretreatments enhance the dendritic spine in the cortical penumbra. (a) Golgi staining images of experimental groups. Scale bar = 10 *μ*m. (b) Quantitative analyses of the spine density of the cortex. Data expressed as mean ± SEM. One-way ANOVA with Tukey's tests, *n* = 7. The WT vs. WT+stroke groups, ^∗∗∗^*P* < 0.0001; the WT+stroke group vs. WT+stroke+TP group and WT+stroke+K group, ^#^*P* < 0.05, ^##^*P* < 0.01; and the WT+stroke+K+TP group vs. WT+stroke+TP group and WT+stroke+K group, ^^^^^*P* < 0.001.

24 hours after cerebral ischemia, we observed considerable reduction of dendritic spine density in the WT+stroke group in comparison with the WT group (Figures 5(a) and (b), ^∗∗∗^*P* < 0.001). In similitude to the WT+stroke group, dendritic spine density in both the WT+stroke+TP and WT+stroke+K groups were increased (^#^*P* < 0.05, ^##^*P* < 0.01). We also found no significant difference in the dendritic spine density between the WT+K and WT groups. With Ki20227 and TP pretreatments, the dendritic spine density of the WT+stroke+K+TP group was significantly increased when compared with both the WT+stroke+TP and WT+stroke+K groups (^^^^^*P* < 0.001).

### 3.6. TP and Ki20227 Pretreatments Upregulate Synaptic Protein Expressions

**Figure 6:**
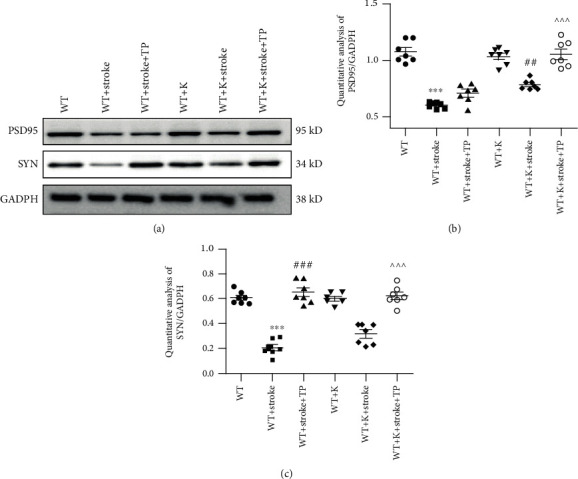
TP and Ki20227 increase synaptic protein expressions. (a) Western blot images of SYN and PSD95 protein expressions of experimental groups. (b) Quantitative analyses of PSD95 protein expression and SYN protein expression. (c) Data were expressed as mean ± SEM. One-way ANOVA with Tukey's tests, *n* = 7. The WT vs. WT+stroke groups, ^∗∗∗^*P* < 0.001; the WT+K vs. WT+stroke+K groups, ^∗∗^*P* < 0.01; the WT+stroke+TP group vs. WT+stroke group, ^###^*P* < 0.001; and the WT+stroke+K+TP group vs. WT+stroke+TP and WT+stroke+K groups, ^^^^^*P* < 0.001.

We used immunoblotting to detect protein levels of dendritic spine synapse markers, such as PSD95 and SYN. Figures 6(a) and 6(b) illustrate PSD95 protein levels, while Figures 6(a) and 6(c) depict SYN protein levels. Both PSD95 and SYN protein expressions were significantly lower in the WT+stroke group than the WT group (^∗∗∗^*P* < 0.001). Compared with the WT+K group, PSD95 and SYN protein expressions were significantly decreased in the the WT+stroke+K group (^∗∗∗^*P* < 0.001). PSD95 protein expression was significantly increased in the WT+stroke+K group when compared with the WT+stroke group (^##^*P* < 0.01). However, there was no significant difference in the SYN expression in the WT+stroke+K group when compared with the WT+stroke group, although no significant difference was discerned between the WT+stroke+TP and WT+stroke+K groups. Regarding SYN protein expression, the WT+stroke+TP group had an upregulated protein expression when compared with the WT+stroke group (^###^*P* < 0.001). In the WT+stroke+K+TP group, PSD95 and SYN expressions were significantly increased in comparison with the WT+stroke+TP, WT+stroke+K, and WT+stroke groups (^^^^^*P* < 0.001).

### 3.7. Ki20227 and TP Activate Akt-Erk Pathway

**Figure 7:**
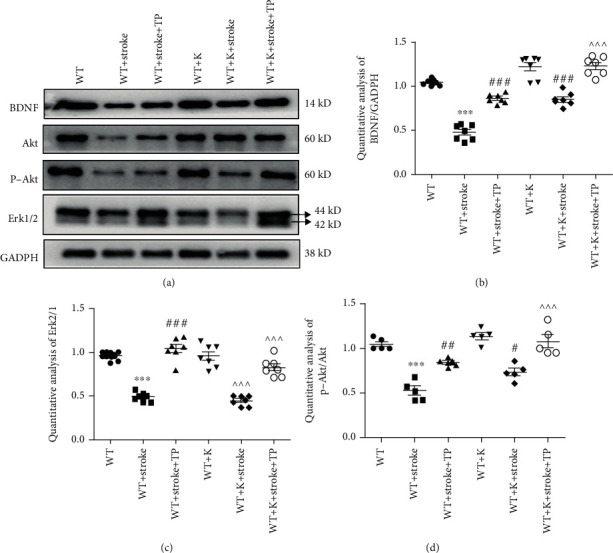
TP and Ki20227 activate the Akt-Erk pathway. (a) Western blot images of BDNF, Akt, p-Akt, Erk1/2, and GADPH of experimental groups. Quantitative analyses of (b) Erk1/2 expression, (c) BDNF expression, and (d) p-Akt/Akt expression. Data were expressed as mean ± SEM. One-way ANOVA with Tukey's tests, *n* = 7. The WT vs. WT+stroke group and WT+K vs. WT+stroke+K group, ^∗∗∗^*P* < 0.001; the WT+stroke group vs. WT+stroke+TP group and WT+stroke+K group, ^#^*P* < 0.05, ^##^*P* < 0.01, ^###^*P* < 0.001; and the WT+stroke+K+TP group vs. WT+stroke+TP and WT+stroke+K groups, ^^^^^*P* < 0.001.

BDNF participates in the proliferation and differentiation of neurons and recovery of brain function after injury. Autophagy and PI3K/AKT or MAPK/Erk1/2 signaling pathways are important downstream pathways that connect BDNF.  Figure 7 illustrates altered expression levels of BDNF, p-Akt/Akt, and Erk1/2 proteins after stroke. BDNF, p-Akt/Akt, and Erk1/2 expressions were significantly decreased after cerebral ischemia (^∗∗∗^*P* < 0.001) and increased in the WT+stroke+TP group in comparison with the stroke group. Compared with the WT+K group, BDNF, p-Akt/Akt, and Erk1/2 expressions were decreased in the WT+stroke+K group. Also, BDNF and p-Akt/Akt expressions were increased in the WT+stroke+K group when compared with the WT+stroke group (^###^*P* < 0.001). Interestingly, BDNF and p-Akt/Akt expressions in the WT+stroke+K+TP group were increased when compared with both the WT+stroke+TP and WT+stroke+K groups (^^^^^*P* < 0.001). Regarding Erk2/1 expression, there was no significant difference between the WT+stroke+TP and WT+stroke+K groups, though it was upregulated in the WT+stroke+K+TP group when compared with both the WT+stroke+K and WT+stroke groups (^###^*P* < 0.001, ^^^^^*P* < 0.001).

### 3.8. Ki20227 and TP Regulate Autophagy Pathway in Stroke Mice

**Figure 8:**
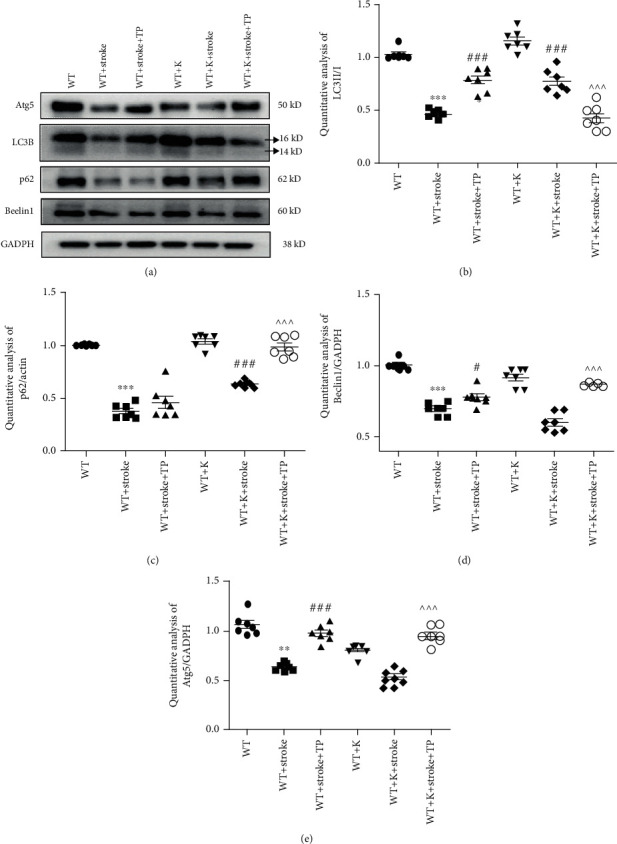
TP and Ki20227 regulate autophagy pathway. (a) Western blot images of Atg5, p62, LC3, Beclin1, and GADPH of experimental groups. Quantitative analyses of (b) p62 expression, (c) LC3II/I expression, (d) Beclin1 expression, and (e) Atg5 expression. Data were expressed as mean ± SEM. One-way ANOVA with Tukey's tests, *n* = 7. The WT vs. WT+stroke and WT+K vs. WT+stroke+K groups, ^∗∗^*P* < 0.01, ^∗∗∗^*P* < 0.001; the WT+stroke+TP and WT+stroke+K groups vs. WT+stroke group, ^#^*P* < 0.05, ^###^*P* < 0.001; the WT+stroke+K+TP group vs. WT+stroke+TP and WT+stroke+K groups, ^^^^^*P* < 0.001.

Autophagy marker protein levels, such as LC3II, Atg5, Beclin1, and p62 were evaluated and shown in Figure 8. Compared with both the WT and WT+K groups, LC3II/I, Atg5, Beclin1, and p62 expressions were significantly decreased in the WT+stroke and WT+stroke+K groups, respectively (^∗∗^*P* < 0.01, ^∗∗∗^*P* < 0.001). Also, in both the WT+stroke+TP and WT+stroke+K groups, the expression ratio of LC3II/I was increased in comparison with the WT+stroke group, and LC3II/I expression was higher in both the WT+stroke+TP and WT+stroke+K groups than the WT+stroke+K+TP group (^###^*P* < 0.001, ^^^^^*P* < 0.001). There were no significant differences in LC3II/I, Atg5, Beclin1, and p62 expressions between the WT+K and WT groups. Concerning Atg5 and Beclin1 protein levels, they were significantly increased in the WT+stroke+TP group when compared with the WT+stroke group (^#^*P* < 0.05, ^###^*P* < 0.001). However, there was no significant difference in the WT+stroke+K group when compared with the WT+stroke group. Further, Atg5 and Beclin1 expressions were increased in the WT+stroke+K+TP group when compared with the WT+stroke and WT+stroke+TP groups (^^^^^*P* < 0.001). Similarly, p62 showed an upregulated expression in both the WT+stroke+K and WT+stroke+K+TP groups when compared with the WT+stroke (^###^*P* < 0.001) and WT+stroke+K groups (^^^^^*P* < 0.001).

## 4. Discussion

**Figure 9:**
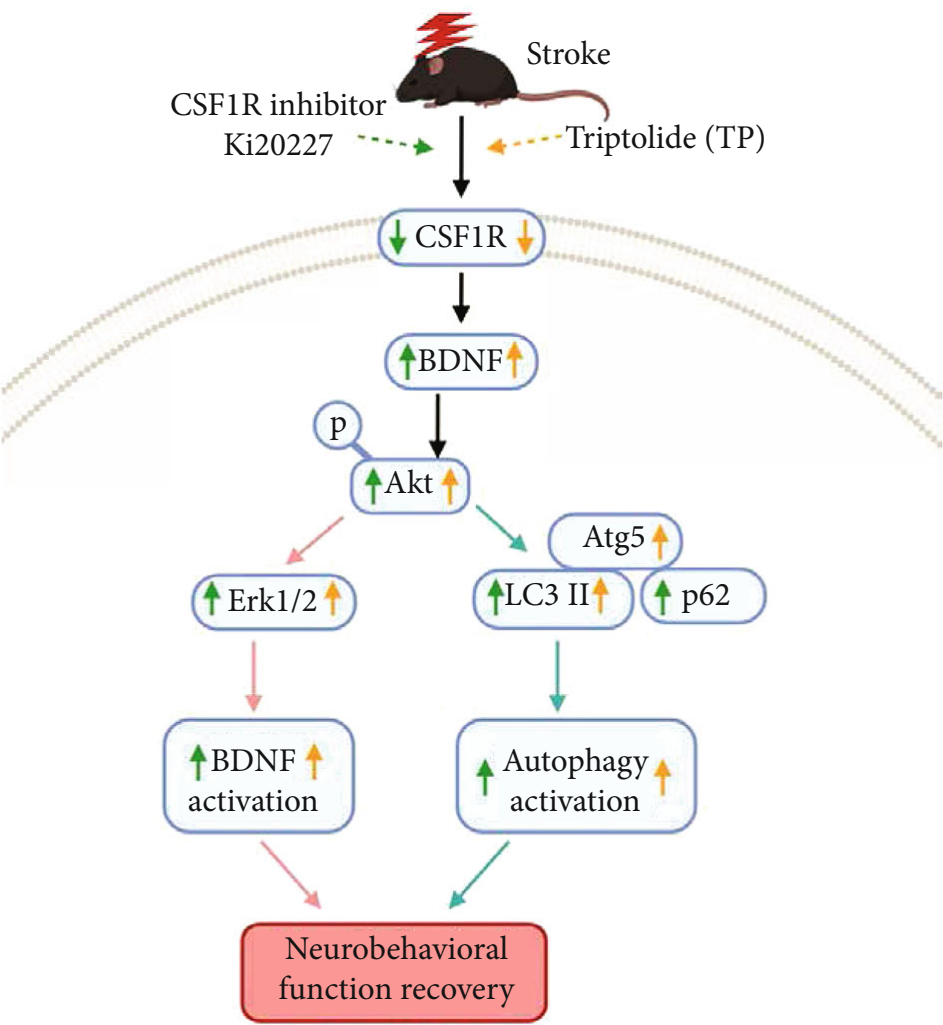
Illustrative mechanism following pretreatments with Ki2027 and TP in mouse model of ischemic stroke. The upregulation of BDNF, Erk1/2, and autophagy protein expressions contributed to neuronal recovery.

An effective medicinal agent that mitigates nerve injury after stroke is of great importance. In this study, we have evidenced that combined Ki20227 and TP pretreatment exert neuroprotective effect in stroke by alleviating neuronal synaptic injury through upregulation of PSD95 and SYN synaptic proteins and dendritic spine density. Also, TP promoted the neuroprotective effect of Ki20227 by inhibiting CSF1R expression and upregulating BDNF, Erk1/2, and autophagy protein expressions in stroke mice. Further, optimized integration of TP and Ki20227 upregulated NeuN expression and downregulated microglia-related Iba1 expression, which in turn enhanced neurobehavioral function (Figure 9).

In this study, we focused on the formation of lesions and the molecular mechanism pertinent to ischemia penumbra in specific brain regions. Photochemical induction method was employed by intraperitoneally injecting Rose Bengal dye and catalyzed by a specific cold light source (560 nm) that caused the formation of monooxygenase free radicals in the local tissue. It is noteworthy that monooxygenase free radicals trigger lipid peroxidation and thrombosis that lodges in the blood vessels to cause vascular occlusion [21]. Besides, the benefit of using the photochemical induction techniques in studying neurological diseases has been previously reported. Such benefits include its ability to focus on a specific brain region while also modifying infarct size by regulating the intensity and duration of the cold light source [22]. The brain region focused in this study was located at 1.75 mm lateral to Bregma, +0.5 mm, left hemisphere. Both the ischemia core and penumbra region were distinguished by TTC staining (Figure 3(b)), with neuronal injury in the ischemia core and normal region being shown in Thy-YFP mice (Figure 3(a)). Injury to the right forelimb motor cortex causes brain function dysfunction and motor coordination imbalance and can be evaluated by rotarod and neurobehavioral score tests [23]. In this study, Ki20227 and TP administrations evidently increased the time mice spent in the rotarod test along with decreased neurobehavioral score and corroborates with a previous report that demonstrated impaired behavior after stroke to be improved following TP administration [11].

TP has shown its neuroprotective features in several central nervous system diseases, including ischemic stroke [11]. Notably, 6-week administration of TP (at a dose of 5 *μ*g/kg) in SD rats attenuated oxidative stress, learning and memory deficits, and neuronal apoptosis that had been caused by chronic cerebral hypoperfusion-induced vascular dementia [24]. Similarly, TP at a dose of 0.2 mg/kg reduced the area of infarction and downregulated NF-*κ*B and astrocyte inflammatory reaction in MCAO [12]. Moreover, TP exerts potent immunosuppressive function and protects neurons by inhibiting microglia activation [25, 26]. Consistent with previous studies, our study showed preadministered TP (0.2 mg/kg) to downregulate microglia-related Iba1 and CSF1R expressions, which in turn inhibited microglia inflammation.

The inhibition of CSF1R signal can exert a neuroprotective role in several diseases [27]. For instance, the CSF1R inhibitor, JNJ-527, inhibited microglial proliferation and modified microglial phenotype while also attenuating tau-induced neurodegeneration to enhance functional improvement in P301S transgenic mice [28]. In a global ischemic injury, the administration of Ki20227 at a dose of 2 mg/g/day for five days culminated in considerable decrements of microglia and neuronal densities [6], thus evidencing that CSF1R inhibition could aggravate ischemic injury. In neurodegenerative disease, CSF1R signaling activates proinflammatory processes through the regulation of CSF1 and IL-34 signaling [29]. It is noteworthy that CSF1 and IL-34 proffer neuroprotection against neurodegeneration by significantly decreasing excitotoxic damage that causes neuronal loss [30]. Thus, therapeutic agents inhibiting CSF1R in the microglia could be promising in the treatment of neurological diseases. In our study, the CSF1R pathway was activated in stroke model mice, with CSF1R and Iba1 protein expressions being upregulated. The individual preadministered Ki20227 and TP curtailed microglia-positive cells via downregulated Iba1 and CSF1R expressions. However, the optimized integration of TP and Ki20227 showed that TP and Ki20227 can inhibit the CSF1R pathway to protect neurons in ischemic stroke.

One of the mechanisms appertain to cerebral ischemic injury treatment could lie in understanding neuronal synaptic plasticity [31]. Cerebral ischemic injury has pronounced an effect on synaptic proteins, as the ultrastructure of synapse is damaged, along with decrement in both synaptic vesicles and synaptic gap [32]. The minimization of neuronal dendritic spine and synaptic plasticity damages after cerebral ischemia is paramount in maintaining neuronal function [33]. Following cerebral ischemia, neuronal axis structures were destroyed, along with depleted dendritic spine density. However, the group that had been pretreated with coadministered TP and Ki20227 showed promotion of upregulated expressions of NeuN, PD95, and SYN in the penumbra area of the cerebral ischemia, thus, evidencing the protectiveness of the pretreated agents against neuronal and synaptic damages.

The neurotrophic BDNF is a key mediator in neuronal survival, differentiation, and plasticity, along with its involvement in the regulation of learning stimulated by synaptic formation [34]. Increasing BDNF production could facilitate the acquisition and retention of motor skills for poststroke rehabilitation by promoting neuroplasticity [35]. In the present study, Ki20227 and TP administration upregulated BDNF protein expression, which we believe was due to the synaptic regulatory function of the CSF1R inhibitor. The upregulation of protein kinase B (Akt) and Raf/mitogen-activated protein kinase (MAPK)/extracellular signal-regulated kinase (Erk1/2) signaling pathways as BDNF downstream factors exert possible protection in stroke [36]. Also, Akt/Erk1/2 pathways promote neurological function, survival rate, and infarct volume enhancements poststroke [37]. In this study, we found pronounced downregulated expressions of p-Akt/Akt and Erk1/2 after cerebral ischemia. However, activated p-Akt/Akt and Erk1/2 expressions were evident in groups that has been pretreated with either TP or Ki20227.

Autophagy, a target pathway of BDNF, is a catabolic process conserved among all eukaryotic organisms. From regulating fundamental metabolic functions inside cells to various diseases, such as aging, cancer, neurodegenerative disorders, and lysosomal disorders, autophagy occurs in any tissue or disease and has become the central regulating point in controlling homeostasis of the human body [38]. The augmented activity of autophagy was evidenced to be involved in synaptic loss [39]. Our study found cerebral ischemia to cause significant curtailment of autophagy, which could have led to synaptic loss. We also found groups that were preadministered Ki20227 to activate autophagy by upregulating the p62 expression. However, the optimized integration of TP and Ki20227 activated autophagy via upregulated expressions of its related proteins (Atg5, Beclin1, and p62).

In summary, our study has evidenced that combined pretreatment with TP and Ki20227 modulates BDNF-Akt and autophagy pathways and confers protectiveness against cerebral ischemia. With several research quarters reporting the involvement of microglia autophagy in the regulation of immune system, inflammatory reaction, and synaptic plasticity [40], our future study would be centered building an autophagy-deficient microglia animal model using transgenic mice and explicate the specific role of autophagy in the microglia after cerebral ischemia.

## Data Availability

Data supporting these study findings are available from the corresponding authors upon reasonable request.

## Abbreviations

CSF1R: Colony-stimulating factor 1 receptor
TP: Triptolide
SYN: Synaptophysin
ROS: Reactive oxygen species
MCAO: Middle cerebral artery occlusion
PBS: Phosphate-buffered saline
ECL: Enhanced chemiluminescence
BDNF: Brain-derived neurotrophic factor
AKT: Protein kinase B
MAPK: Raf/mitogen-activated protein kinase
ERK: Extracellular signal-regulated kinase.
